# A methodological approach to the air-sea energy fluxes data collection and analysis at the tropical coastal ocean

**DOI:** 10.1016/j.mex.2018.05.003

**Published:** 2018-05-17

**Authors:** Yusri Yusup, John Stephen Kayode, Abbas F.M. Alkarkhi

**Affiliations:** a Environmental Technology, School of Industrial Technology, Universiti Sains Malaysia, USM, 11800, Pulau Pinang, Malaysia; b Centre for Marine & Coastal Studies (CEMACS), Universiti Sains Malaysia, Pulau Pinang, Malaysia; c Malaysian Institute of Chemical & Bioengineering Technology, Universiti Kuala Lumpur, 78000, Melaka, Malaysia

**Keywords:** R statistical analysis of eddy covariance data, R statistical software, Microclimate variables, Tropical coastal ocean, Energy fluxes, Eddy covariance

## Abstract

The southern South China coastal oceans within the South East Asian region are much lacking in the perception of the surface energy budget and evaporation over the ocean waters in response to climatic changes. The eddy covariance method was used to measure the energy fluxes, microclimate variables, and surface water temperature from November 2015 to October 2017 at the Straits of Malacca, South China Sea; Pulau Pinang, Malaysia, situated at latitude 5°28′06″N, and longitude 100°12′01″E. This work focused on the methodological approach to the air-sea energy fluxes data collection and analysis. In this regard, the method applied for the direct measurements and analysis of energy fluxes and other meteorological parameters in the site is considered and reported.

• The paper summarizes the analysis of energy fluxes, microclimate variables, and surface water temperature data in a tropical coastal ocean station using the eddy covariance method.
• The methodological approach illustrates the method of analysis applied in this study which can be compared and used for similar studies in other places.
• The reproducible data analysis technique matches similar comparative methods such as Matlab and Python.

## Graphical abstract

**Figure:**
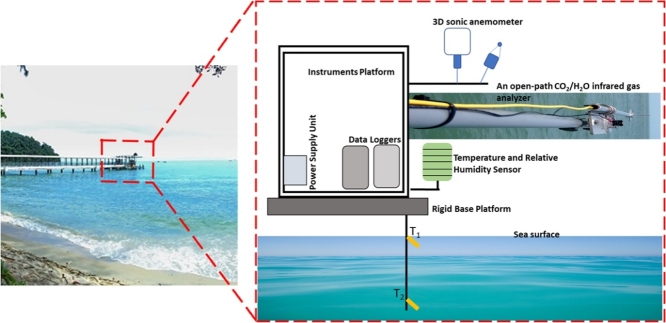

Specifications table

**Table:** 

Subject area	• Earth and Planetary Sciences • Environmental Science
More specific subject area	*Atmospheric Science*
Method name	*R statistical analysis of eddy covariance data*
Name and reference of original method	*R programming, eddy covariance processing using EddyPro version 6*
Resource availability	*Data and the data analysis programming codes*

## Method details

**Fig. 1:**
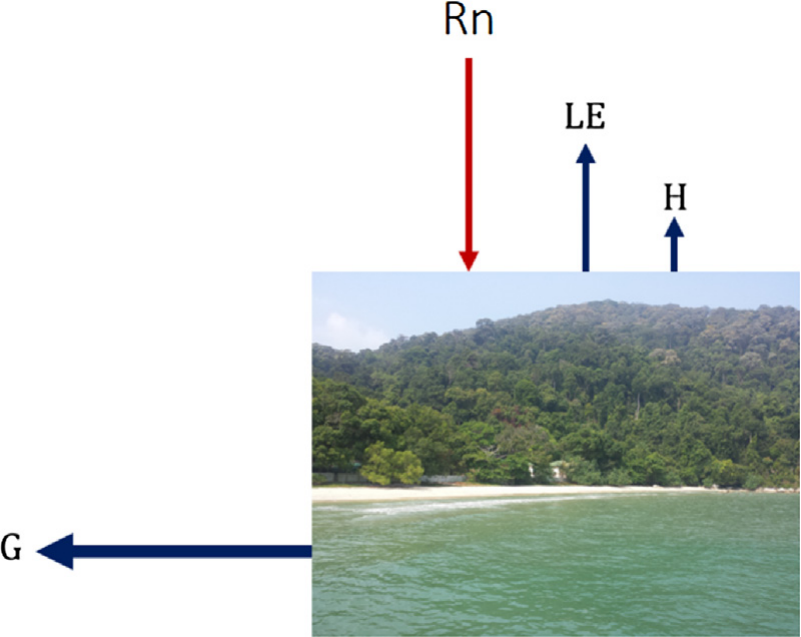
The energy budget at the tropical coastal ocean station named as the Muka Head station, Pulau Pinang, Malaysia, in the southern South China Sea (5°28′6″N, 100°12′1″E).

Understanding of the exchange of energy at the Earth’s surface is necessary for the improvement of regional weather forecast and global climate models. The main source of energy come from the Sun in the form of the global radiation symbolized by *R_G_*. The amount of energy absorbed by the ocean is denoted as the net radiation (*R_N_*). Part of the energy is stored inside the ocean as the residual, G. The ocean emitted some energies in the form of evaporation as latent heat (*LE*) and sensible heat (*H*) fluxes, to the atmosphere [1]. Assuming horizontal homogeneity, the surface energy balance, (Fig. 1) of ocean could be expressed as,

(1) *R_N_ *= *LE *+ *H *+ *G*

where *R_N_R_n_* and *G* are positive when the ocean absorbed the energy. However, *LE* and *H* are positive when the energy is released from the ocean. The unit of measurement of the fluxes is in W m^−2^ [2].

From Eq. (1), energy balance closure can be used to give an overview on the validity of surface layer measurements using the Eddy Covariance (EC) system of measurements. Though there is no perfect energy balance equation, it is understandable that energy balance at the Earth’s surface could not be closed for many experimental datasets from varied surfaces, e.g., oceanic and terrestrial surfaces [1].

The ratio between *LE* and *H* fluxes could be computed to evaluate the energy balance closure (or EBC in the form of fraction or percentage). The absolute energy residual (*R_es_*, W m^−2^) was calculated using Eq. (2). *R_es_* is added to (1) to get a complete balance equation for the energy as shown in (3) [1,2]. G is calculated from the integration of the changes in the underwater temperature profile with time.

(2) *R_es_ *= *R_N_ *− *H *− *LE − G*

(3) *R_N_ *= *R_es_ *+ *H *+ *LE + G*

**Fig. 2:**
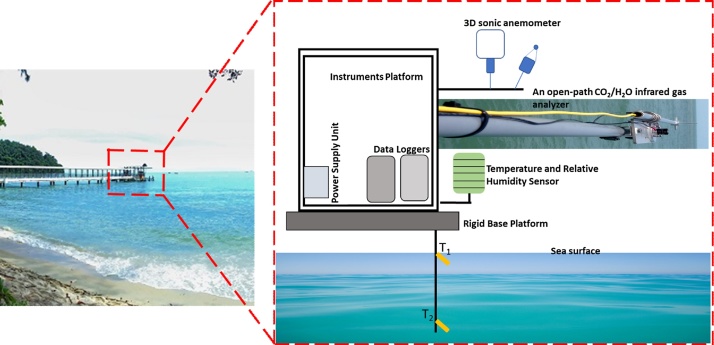
Location of the study site named as the “Muka Head” Station (5°28′6″N, 100°12′1″E) while the right panel is zoomed-out view of the site showing the Instruments Platform together with the sensors and the eddy covariance system.

The fluxes were measured using an eddy covariance system, which was installed on a stable stainless-steel platform extending a pre-existing pier so that the system would be directly over the tropical coastal ocean. The eddy covariance system included a 3-D sonic anemometer (model 81000 V, RM Young, USA) and an open-path CO_2_/H_2_O gas analyzer (model LI-7500 A, LI-CO−COR, Inc., USA) installed 4.1 m above the sea water surface, (Fig. 2). The gas analyzer and sonic anemometer were factory-calibrated before deployment. A data logger (model LI-7550 Analyzer Interface Unit, LI-COR, Inc., USA) was used to record the eddy covariance data at a frequency of 20 Hz. The sonic anemometer was also used to measure wind speed (U) and wind direction (WD). The flux data was averaged in 30-min blocks. The flux movement convention used is, negative flux value indicating downward-moving flux while positive value indicating upward-moving flux.

To complement the eddy covariance system, the “Biomet” system of slow-response sensors measured the microclimate variables, the atmospheric temperature (T_A_) and relative humidity (RH) sensor (model HMP155, Vaisala, Finland; the accuracy of T_A_ is ± 0.7 °C and the accuracy of RH is ±1.7%), a pyranometer (R_G_) (model LI-200SL, LI-COR, Inc., USA; error was <5%), and a net radiometer (R_N_) (model NR LITE 2, Kipp & Zonen, Inc., USA; sensitivity was 13.6 μV W^–1^ m^–2^). Sea water temperatures (T_S_) at depths of 0.5 m (T_S1_) and 2.5 m (T_S2_) were measured using two thermistors (LI-COR, Inc., USA) with the accuracy of ± 1 °C. Since T_S1_ was positioned near the water surface, the temperature measurements were assumed to be the sea water surface temperature. An additional data logger recorded the Biomet data (model 9210b Xlite, Sutron Corporation, USA) at a sampling frequency of 1 min and averaged in 30-min blocks. All slow-response sensors were factory-calibrated before installation. Cumulative 15-min precipitation data (Davis Vantage Pro2 Plus, Davis, USA) was retrieved from a nearby weather station IPENANGP2 (5°28 4′'N, 100°17′25′'E).

The EddyPro Version 6 software (LI-COR, Inc., USA) was used to process the raw 20-Hz data [3]. The software was used to filter the raw datasets using methods described by [4] specifically, the following operations were carried, i.e., amplitude resolution, spike removal, dropouts, absolute limits in addition to skewness and kurtosis. The sonic anemometer was subjected to two-fold rotations technique for the tilt correction to guarantee that the mean vertical wind speed is zero. Footprint estimation as described by [5] was also employed.

### Method validation

*1. Quality Control:* The fluxes are quality-flagged in the EddyPro software in which the final flag outputs was based on a combination of two tests, each providing a partial standard. The two tests are the steady-state test and developed-turbulence conditions tests. The final standard outputs with the value “0” indicates the best quality flux data, “1” means good quality fluxes while “2” means bad quality which was subsequently removed from the data. The classification of energy fluxes flagging was developed after the second discussion in the CarboEurope workshop on the quality assurance and quality control of EC measurements [6, 7]. In addition, the ratio of the standard deviation for the vertical component of the wind speed, *σ_w_* (m s^−1^) and the friction velocity, u_*_(m s^−1^), i.e., *σ_w_*/u_*_ was found to hold only for wind directions 0° to 90°, which was typically from the surface of the ocean with a homogeneously flat surface. This ratio did not hold for other directions, i.e., typically for rough surfaces from 90° to 360° and thus, flux data from these quadrants were discarded. The percentage of data retained, after following the quality-controlled procedures, is 21% for LE and H. The data was not filled using any data-gap method.

**Fig. 3:**
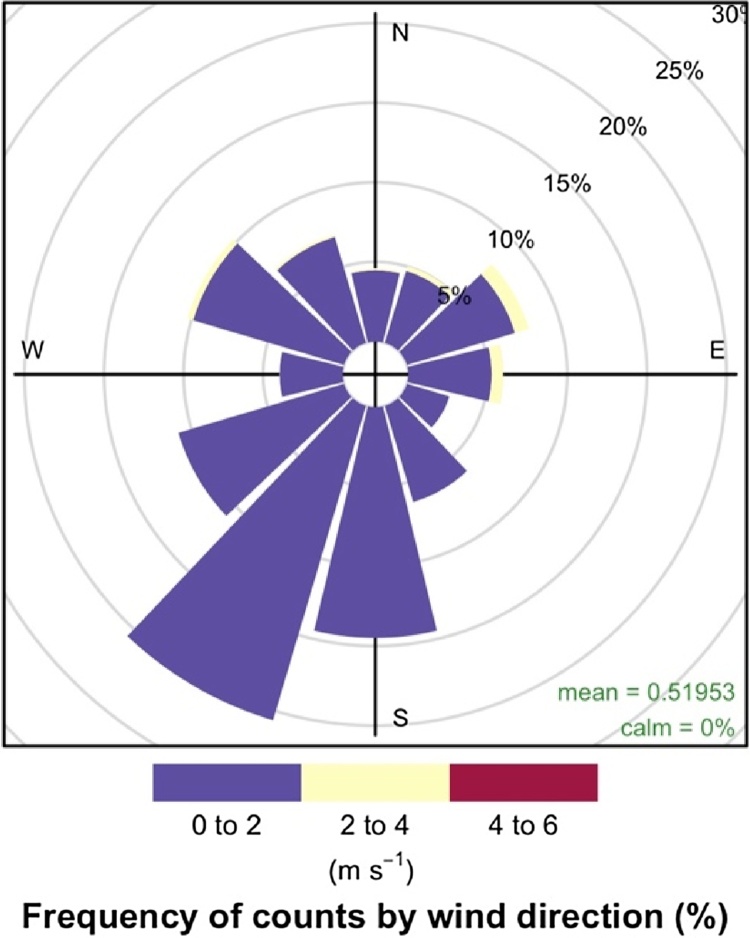
Wind rose obtained for the period from November 2015 to October 2017 with the frequency count in percentage.

*2. Wind Speed*: One of the physical drivers of energy fluxes is the Wind Speed (WS) [8]. The average WS in the tropical coastal ocean location was mostly light, with an average of 0.520 m s^−1^ throughout the study period. As shown in Fig. 3, most of the high WS values of between 2 m s^−1^ and 4 m s^−1^ were north-easterly (0° to 90°), which was blowing from the sea direction and finally used in further analysis. The WS value observed from the inland along the south through to the west directions to the station, i.e., between 90° and 360° were discarded during the analysis due to poor-quality flags and deviations from the ratio 1.3, as mentioned above. However, the wind rose show that winds were evenly-distributed among the four quadrants, which suggests that fluxes were well-represented for all seasons throughout the study period.

3. *Turbulent heat fluxes:* The latent heat (*LE*) and sensible heat (*H*) fluxes in this study location were relatively low, which ranged from −36 W m^−2^ to 130 W m^−2^ and −12 W m^−2^ to 24 W m^−2^, respectively. The low values recorded could be perhaps due to the weak winds velocities of this location. The average half-hourly *LE* showed a vertical positive flux of water vapor with 11.67 W m^-2^ value. *H* was much lower than *LE*, which recorded at the half-hourly mean of 1.56 W m^−2^. The Bowen ratio (the ratio of *H*/*LE* = 0.13) is equivalent to the global ocean estimate.

*4. Underwater temperature:* In this study, underwater temperature (°C) trends at two levels were measured. *T_S1_* is the temperature near the surface while *T_S2_* is the temperature nearer to the seabed. These two underwater thermistors showed that the temperatures varied greatly between 27 °C and 33 °C. *T_S1_* was always higher than *T_S2_*, with mean temperatures at 30.85 °C and 30.27 °C, respectively. This is because the thermistor that measured the temperature at *T_S1_* is sometimes exposed to the atmosphere, due to low tides, and thus recorded a higher temperature in comparison to *T_S2_* located in the ocean water. There were some data gaps for *T_S1_* between the month of August and mid-September. This was caused by the breakdown of thermistor near the water surface, giving super extreme values and so the data was discarded. Generally, *T_S1_* and *T_S2_* did not vary significantly but they showed large changes in the temperatures diurnally. However, there was no difference in the water temperatures at the two depths.

*5. Global and net radiations:* The half-hourly ranges of *R_G_* and *R_N_* were 0 W m^−2^ to 1020 W m^−2^ and -130 W m^−2^ to 990 W m^−2^, respectively. The recorded global *R_G_* and net *R_N_* radiations during the reporting periods recorded peak values during the middle of March, for both *R_G_* and *R_N_*, averaged at 217 W m^−2^ and 163 W m^−2^, respectively. This situation is an indication that the tropical coast of southern South China Sea received the maximum amount of solar radiation, possibly due to the extreme heat wave from the sun along the tropical equatorial belt in this month.

*6. Air temperature and relative humidity:* Fluctuations in the air temperature recorded between 22 °C to 32 °C throughout the study period. The day time temperature, obtained was considerable higher than the night time temperature. Differences in the temperatures was apparent particularly during precipitation in the early hours of the day and at night time. The last microclimate variable measured at the station is the relative humidity (RH) expressed in percentage, %, which ranged from 50% to 100%. This range is typical of tropical locations.
